# Repetitive Negative Thinking is Associated With Depression and Feeding Status at 3 and 6 Months Postpartum: Retrospective Study

**DOI:** 10.31083/AP38795

**Published:** 2025-02-28

**Authors:** Chunfeng Xing, Guoxin Li, Guangqing Zhang, Yaxin Liu, Meirong Yan, Guilin Liu

**Affiliations:** ^1^Department of Nursing, Shenzhen Guangming Distract People’s Hospital, 518106 Shenzhen, Guangdong, China; ^2^Department of General Surgery, NanFang Hospital, 510100 Guangzhou, Guangdong, China; ^3^College of Nursing, NanFang Hospital, 510100 Guangzhou, Guangdong, China; ^4^Department of Obstetrics, Shenzhen Guangming District People’s Hospital, 518106 Shenzhen, Guangdong, China

**Keywords:** mental health, repetitive negative thinking, depression, breastfeeding, postpartum depression, maternal wellbeing

## Abstract

**Objective:**

To investigate the association between repetitive negative thinking and depression as well as feeding status at 3 and 6 months postpartum.

**Method:**

One hundred and twenty-eight pregnant women recruited by the hospital from January 2020 to June 2022 were selected for the study. General demographic data of pregnant women, the multiple Persistent Thinking Questionnaire (PTQ), the Edinburgh Postnatal Depression Scale (EPDS) at 3 and 6 months postpartum, and breastfeeding status were collected. According to PTQ scores, the pregnant women were divided into high subgroup (scores ≥30) and low subgroup (scores <30). Intergroup comparisons of continuous variables following a normal distribution were performed using the *t*-test, while categorical data were analyzed using the χ^2^ test. Spearman correlation analysis was conducted to examine the relationship between PTQ, EPDS, and breastfeeding status.

**Results:**

EPDS scores were higher in the high group than in the low group at 3 and 6 months postpartum (*p* < 0.001). The breastfeeding rates in the high group were lower than that in the low group at 3 and 6 months postpartum (*p* < 0.001). Higher PTQ scores were associated with postpartum depression at 3 and 6 months (r = 0.379, *p* < 0.001; r = 0.358, *p* < 0.001) and lower breastfeeding rates (r = –0.346, *p* < 0.001; r = –0.353, *p* < 0.001).

**Conclusions:**

Higher PTQ scores are associated with increased postpartum depression and reduced breastfeeding rates at 3 and 6 months postpartum, suggesting that repetitive negative thinking may be related to postpartum mental health and feeding outcomes.

## Main Points:

1. Repetitive negative thinking, measured by the Persistent Thinking Questionnaire (PTQ), significantly related to postpartum depression (Edinburgh Postnatal Depression Scale (EPDS) scores) and breastfeeding outcomes at 3 and 6 months, emphasizing its role in maternal well-being.

2. The PTQ proves effective as a screening tool for assessing the likelihood of postpartum depression and breastfeeding difficulties, highlighting its potential to guide early intervention strategies.

3. Addressing repetitive negative thinking during pregnancy can potentially improve maternal mental health stability and enhance breastfeeding rates, underscoring the importance of proactive support measures.

## 1. Introduction

Epidemiologic report [1] indicates that postpartum depression often occurs 2–6 weeks after delivery, with a global incidence of about 11–20%. This condition not only impacts the physical and mental health of mothers but also has long-term negative effects on infant growth and development. 50–80% of women might experience transient mild depression during pregnancy or after delivery, 10–15% of women may experience more severe symptoms of postpartum depression, while 0.1–0.2% of women may develop postpartum psychosis [2]. These findings highlight the severity of postpartum depression as a global public health issue, emphasizing the importance of early detection and intervention for the effective prevention and control of postpartum depression.

While breastmilk remains the primary source of nutrition for infants and is vital for their healthy growth and development during the first six months of life, the presence of postpartum depression among mothers can significantly affect breastfeeding practices and, consequently, infants’ nutritional intake and overall well-being [3]. Additionally, breastfeeding can enhance communication between mothers and infants, promote efficient uterine contractions, and aid in the discharge of Lochia, thereby accelerating postpartum recovery [4]. While breastfeeding has been clinically recognized as advantageous, it still faces issues such as low exclusive breastfeeding rate and short feeding time [5]. According to statistics [6], China’s breastfeeding problem is particularly severe, with around 70% of mothers experiencing the difficulties. Therefore, improving the breastfeeding rate and prolonging feeding time have become top priorities for the clinical health of both mothers and infants.

Repetitive negative thinking refers to non-constructive, intrusive, and repetitive thought pattern. It is often caused by stressful life events such as unemployment, widowhood, divorce, and serious illnesses. In the past, repetitive negative thinking has been clinically associated with mental health, particularly anxiety and depression in adults, both pregnant and non-pregnant females. There is a strong relationship between Repetitive negative thinking and depression and anxiety [7]. Moulds ML *et al*. [8] has found a significant correlation between repetitive negative thinking, depression and anxiety during pregnancy and postpartum.

A strong link has been found between repetitive negative thinking and mental health and feeding status in the six months following childbirth [9]. This means that postpartum women may experience negative thought patterns, which further affects their mental health status and how and how often they feed their babies. However, there is no clinical report on predicting postpartum depression and breastfeeding by measuring the levels of prenatal repetitive negative thinking in mothers. This paper analyses the correlation between repetitive negative thinking and the postpartum depression and breastfeeding. The aim is to provide references for the development of countermeasures as early as possible and guidance for clinical practice.

## 2. Information and Methods

### 2.1 General Information

A total of 128 pregnant women were recruited from the hospital between January 2020 and June 2022 for this study. The study was approved by the hospital’s Ethics Committee and all participants provided informed consent. Inclusion criteria: ① Mothers with single pregnancy; ② Mothers age 18 to 35 years old; ③ Mothers have full-term pregnancy; ④ Mothers have normal maternal cognitive function; ⑤ Mothers sign the maternal informed consent. Exclusion criteria: ① Maternal breast dysplasia, nipple inversion, mastitis, and other conditions that affect breastfeeding; ② Postpartum drug use should be avoided while breastfeeding; ③ Incomplete clinical data or missing/incomplete questionnaires should be addressed; ④ Pregnant women with complications or damage to vital organs such as the heart, liver, kidneys; ⑤ Infants with congenital diseases; ⑥ The mother has a history of prior mental illness or already suffers from a mental illness.

### 2.2 Methods

(1) General demographic data of all mothers were collected when they were hospitalized, including age, body mass index, education, occupation, pregnancy, delivery, adverse pregnancy history, pregnancy complications, mode of delivery, monthly household income, marital status and history of mental illness.

(2) The Multiple Persistent Thinking Questionnaire (PTQ) [10] was selected prenatally to measure the levels of repetitive negative thinking of all mothers. PTQ was mainly used to assess the repetitive negative thinking of mothers. The scale contains fifteen items, covering the content of the participant’s own experiences. Usually when it comes to the negative experiences or problems, the scale was rated on a 5-point Likert scale, with options ranging from “never” to “almost always” respectively given 0–4 points, out of 60, and the higher the score, the greater the frequency of repetitive negative thinking and the greater the impact of repetitive negative thinking. The scale demonstrated good reliability and validity with a Cronbach’s alpha coefficient of 0.92 and a half-reliability coefficient of 0.85.

(3) At 3 and 6 months postpartum, the Edinburgh Postnatal Depression Scale (EPDS) [11] was used to measure depression occurrence in mothers. The EPDS assesses whether mothers suffer from depression and consists of ten items covering self-blame, fear, sadness, mood, insomnia, and pleasure. Each item can be rated on a 4-point Likert scale, with options ranging from ‘never’ to ‘always’ assigned a score of 0–3 out of a possible 30 A score of 13 points or higher is defined as depression [12], with a higher score indicating a greater risk of developing depression. The scale’s Cronbach’s alpha coefficient was 0.87, and the folded-in reliability coefficient was 0.88, indicating good reliability.

(4) All mothers were observed for their breastfeeding status, i.e., whether they had been breastfeeding all the time at 3 and 6 months postpartum.

### 2.3 Observation Indicators

The PTQ scores of all mothers were observed and mothers were grouped according to their scores. Clinical data and EPDS scores at 3 and 6 months postpartum were compared between the two groups. The breastfeeding rates were also analysed. Using Spearman correlation analysis to analyse the correlation between PTQ and depression, as well as breastfeeding.

### 2.4 Statistical Analysis 

The data were analysed using SPSS 25.0 statistical software (IBM Corporation, Armonk, NY, USA). Normality was tested using the Shapiro-Wilk test. Data that conformed to normal distribution were expressed in ($\bar{x}$ ± s), and the *t*-test was used to compare the two groups. Categorical data are only described in the form of n (%), and the χ^2^ test was selected. The correlation was calculated using Spearman correlation coefficient. A statistically significant difference was indicated by *p* < 0.05.

## 3. Results

### 3.1 Status of PTQ Scores

This paper included a total of 128 women with PTQ scores (26.56 ± 3.11). Of these, 78 women with scores <30 were included in the low subgroup, while the remaining 50 women with scores ≥30 were included in the high subgroup.

### 3.2 Comparison of Clinical Data between Groups

There was no difference between the high group compared to the low group in all clinical data (*p* > 0.05). Details are shown in Table 1.

**Table 1.** **Comparison of clinical data between groups (n = 128)**.

Clinical information		n	High scoring groups (n = 50)	Lower scoring groups (n = 78)	*p*
Age (years)	>30	72 (56.25%)	29 (58.00%)	43 (55.13%)	0.749
	≤30	56 (43.750%)	21 (42.00%)	35 (44.87%)	
BMI (kg/m^2^)	>25	64 (50.00%)	23 (46.00%)	41 (52.56%)	0.469
	≤25	64 (50.00%)	27 (54.00%)	37 (47.44%)	
Education	High school and below	69 (53.91%)	25 (50.00%)	44 (56.41%)	0.478
	College and above	59 (46.09%)	25 (50.00%)	34 (43.59%)	
Careers	Incumbency	77 (60.16%)	31 (62.00%)	46 (58.97%)	0.733
	Out of Work	51 (39.84%)	19 (38.00%)	32 (41.03%)	
Number of pregnancies (times)	>2	75 (58.59%)	30 (60.00%)	45 (57.69%)	0.796
	≤2	53 (41.41%)	20 (60.00%)	33 (42.31%)	
Mode of childbirth	Natural Childbirth	48 (37.50%)	17 (34.00%)	31 (39.74%)	0.513
	Cesarean Section	80 (62.50%)	33 (66.00%)	47 (60.26%)	
Number of childbirth (times)	>2	47 (36.72%)	14 (28.00%)	33 (42.31%)	0.101
	≤2	81 (63.28%)	36 (72.00%)	45 (57.69%)	
History of adverse pregnancies	Yes	53 (41.41%)	16 (32.00%)	37 (47.44%)	0.084
	No	75 (58.59%)	34 (68.00%)	41 (52.56%)	
Complications of pregnancy	Yes	56 (43.75%)	22 (44.00%)	34 (43.59%)	0.964
	No	72 (56.25%)	28 (56.00%)	44 (56.41%)	
Monthly Household Income (RMB)	>5000	75 (58.59%)	33 (66.00%)	42 (53.82%)	0.173
	≤5000	53 (41.41%)	17 (34.00%)	36 (46.15%)	
Marriage status	Married	105 (82.03%)	39 (78.00%)	66 (84.61%)	0.342
	Unmarried/Divorced	23 (17.97%)	11 (22.00%)	12 (15.39%)	
History of mental illness	Yes	37 (28.91%)	18 (36.00%)	19 (24.36%)	0.156
	No	91 (71.09%)	32 (64.00%)	59 (75.64%)	

BMI, Body Mass Index.

### 3.3 Changes in EPDS Scores at Different Time Points in Each Group

EPDS scores were higher in the high group than in the low group at 3 and 6 months postpartum (*p* < 0.05). Details are shown in Table 2.

**Table 2.** **Changes in EPDS scores at different time points in each group (score)**.

Groups	3 months postpartum	6 months postpartum
High score group (n = 50)	15.87 ± 4.09	14.52 ± 4.12
Low score group (n = 78)	12.81 ± 3.86	11.67 ± 3.80
*p*	<0.001	<0.001

EPDS, Edinburgh Postnatal Depression Scale.

### 3.4 Breastfeeding Rates at Different Time Points in Each Group

Breastfeeding rates were lower in the high group than in the low group at 3 and 6 months postpartum (*p* < 0.05). For details, see Table 3.

**Table 3.** **Breastfeeding rates at different time points in each group [n (%)]**.

Group	3 months postpartum	6 months postpartum
High score group (n = 50)	17 (34.00)	21 (42.00)
Low score group (n = 78)	54 (69.23)	60 (76.92)
*p*	<0.001	<0.001

### 3.5 Correlation between PTQ and EPDS, Breastfeeding

Table 4 shows a significant correlation between PTQ, postpartum depression (EPDS), and breastfeeding. Specifically, PTQ was positively correlated with EPDS scores at 3 and 6 months postpartum (r = 0.379 and 0.358, respectively; both *p* < 0.001), indicating that higher levels of repetitive negative thinking are associated with more severe postpartum depression symptoms. Meanwhile, PTQ was negatively correlated with breastfeeding rates at 3 and 6 months postpartum (r = –0.346 and –0.353, respectively; both *p* < 0.001), suggesting that higher repetitive negative thinking is linked to lower breastfeeding rates. These findings indicate that maternal repetitive negative thinking may increase the risk of postpartum depression and negatively impact breastfeeding continuity.

**Table 4.** **Correlation between PTQ and EPDS, breastfeeding**.

PTQ	EPDS at 3 months postpartum	EPDS at 6 months postpartum	Breastfeeding at 3 months postpartum	Breastfeeding at 6 months postpartum
r	0.379	0.358	–0.346	–0.353
*p*	<0.001	<0.001	<0.001	<0.001

PTQ, The Multiple Persistent Thinking Questionnaire.

## 4. Discussion

Postpartum depression is an abnormal psychological condition that can lead to mental vulnerability in severe cases. Mothers with postpartum depression constantly focus on the causes and effects of postpartum events, negative emotions, and ineffective coping mechanisms, which can hinder recovery after delivery [13, 14, 15, 16, 17]. According to a study by Ho CSH *et al*. [18], repetitive negative thinking increases the risk of experiencing a depressive episode. The conclusion drawn above is consistent with the findings of the referenced paper. Specifically, the EPDS scores of the high group were higher than those of the low group at 3 and 6 months postpartum, indicating that the risk of postpartum depression increases with the frequency of repetitive negative thinking, which can have more serious and far-reaching consequences. Repetitive negative thinking predicts the onset of new episodes and the maintenance of existing symptoms of depression and is associated with reduced treatment response [19]. Similarly, Moulds ML *et al*. [20] found that repetitive negative thinking was associated with depression, anxiety and other unhelpful cognitive processes in the postnatal period, as well as with poor infant responsiveness. In terms of that, repetitive negative thinking is a cognitive susceptibility factor for depression. After experiencing a negative life event, such as giving birth, a woman’s hormones and emotions undergo changes. Additionally, the new challenges and pressures that come with caring for a newborn can exacerbate these changes and pressure. Mothers may experience self-reproach, helplessness, and despair, as well as negative emotions due to repetitive thinking. This can activate negative cognitive biases including negative memories, metacognition and so on. All of the aforementioned negative emotional states can cause individuals to think in a one-sided and negative manner. This can reduce maternal problem-solving and coping abilities, significantly increasing the risk of postpartum depression [21, 22, 23]. This study replicates the findings of Hirsch CR *et al*. [24], who found a positive correlation between repetitive negative thinking, depression, and anxiety during the perinatal period. Edge D *et al*. [25] also concluded that anxiety and depression are risk factors for repetitive negative thinking, and reducing repetitive negative thinking can reduce anxiety and depression.

Strahm AM *et al*.’s [9] study shows that women with lower levels of repetitive negative thinking have longer breastfeeding durations. This conclusion is consistent with the findings of this paper. The breastfeeding rate of the high group was lower than that of the low group at 3 and 6 months postpartum. The data indicate that there is a negative correlation between the frequency of repetitive negative thinking and the rate of breastfeeding. PTQ and breastfeeding exhibited a negative correlation, indicating a close relationship between repetitive negative thinking and breastfeeding. Prediction during the prenatal period is of higher value and can provide guidance for subsequent development of relevant interventions in advance. Negative thinking can have a direct impact on a mother’s daily diet and nutritional intake. This, combined with loss of appetite and depressed mood, can lead to a reduction in food intake, particularly of nutritious foods. As a result, the mother’s nutritional status may be affected, which can further impact the secretion and quality of breast milk, ultimately having a negative effect on the baby’s health. Furthermore, repetitive negative thinking may affect the functioning of the hypothalamus and pituitary gland, leading to inhibited prolactin production and a significant reduction in lactation [26]. A previous clinical study [26] also found that mothers with a strong tendency towards repetitive negative thinking were less responsive to infant behaviour. This demonstrates the importance of measuring repetitive negative thinking during antenatal care in predicting breastfeeding outcomes. Such measurements can inform the development of relevant measures in the early stages.

However, there are still some limitations in this article, such as the small number of research subjects and the short 6-month study duration, which results in a smaller change score of PTQ. To improve the accuracy of the test results, more subjects can be included in the following clinical study and the follow-up time can be extended.

## 5. Conclusions

In conclusion, the study found that repetitive negative thinking is highly correlated with depression and feeding status at 3 and 6 months postpartum. These results can be used to develop early countermeasures to stabilize maternal psychological state and improve breastfeeding rate.

## Availability of Data and Materials

Data to support the findings of this study are available on reasonable request from the corresponding author.
